# Advancing Pyoderma Gangrenosum Management With a Multidisciplinary Approach

**DOI:** 10.7759/cureus.77879

**Published:** 2025-01-23

**Authors:** Martena Grace, Amir A Estil-las, Camelia Arsene

**Affiliations:** 1 Internal Medicine, Ross University School of Medicine, Miramar, USA; 2 Internal Medicine, St. Joseph Mercy Oakland Hospital, Pontiac, USA

**Keywords:** autoimmune, inflammation, neutrophillic dermatosis, pyoderma gangenosum, skin infections, ulcer

## Abstract

Pyoderma gangrenosum (PG) is a rare neutrophilic dermatosis characterized by aseptic, non-infectious skin lesions. This report details a case of PG in a 49-year-old woman with multiple comorbidities and limited access to healthcare resources. In the absence of standardized treatment protocols, the case illustrates the successful application of a multidisciplinary therapeutic approach combining surgical intervention with medical management using corticosteroids and immunomodulators. The report includes visual documentation of the treatment process and disease progression, providing a comprehensive timeline of clinical outcomes. Furthermore, it highlights the challenges of managing PG in resource-constrained settings, emphasizing the importance of consistent monitoring and proactive intervention to prevent complications.

## Introduction

Pyoderma gangrenosum (PG) is a rare inflammatory skin condition characterized by painful, non-infectious ulcers that can spread to surrounding tissues. It falls under the category of neutrophilic dermatoses (ND), which involves sterile lesions caused by neutrophil-driven inflammation [1].

Individuals with PG often experience painful skin lesions that may develop into open sores, commonly appearing on the arms, neck, lower limbs, and genitals. Other symptoms can include skin discoloration, fever, and joint pain. PG is also commonly associated with rheumatoid arthritis (RA), ulcerative colitis, and Crohn's disease.

The prevalence of PG is estimated to be 5.8 cases per 100,000 adults, with a higher prevalence among women [2]. While the precise pathophysiology remains unclear, it is thought to result from a complex interplay of factors, including abnormal neutrophil activity, T-cell involvement, inflammasome activation, keratinocyte apoptosis, and genetic factors.

Currently, there are no established international guidelines for treating PG. However, studies and case reports have shown symptom improvement or resolution through various therapies. These include wound care, steroids, and immunomodulators [3].

## Case presentation

We present the case of a 49-year-old woman with a medical history of RA, primary hypertension, type 2 diabetes mellitus (T2DM), class 3 obesity, seizure disorder, and hypercholesterolemia. She initially presented with gradually enlarging, red, itchy papules on her left shoulder, which began to drain and develop pustules, eventually progressing to an abscess with overlying skin necrosis.

Due to a delay in initiating treatment in March 2024, the patient was admitted to the hospital, where consults were placed for dermatology, internal medicine, infectious disease, surgery, and wound care. A biopsy resulted in skin and subcutaneous soft tissue ulceration, dense neutrophilic infiltrates, and gangrenous necrosis confirming a diagnosis of PG with necrosis extending to the muscular fascia. There were no signs of infection; her WBC was within the reference range, and wound cultures showed no bacterial growth. An X-ray of the left shoulder showed no signs of osteomyelitis. The patient completed an initial course of empiric antibiotics, cefepime, and vancomycin.

**Figure 1:**
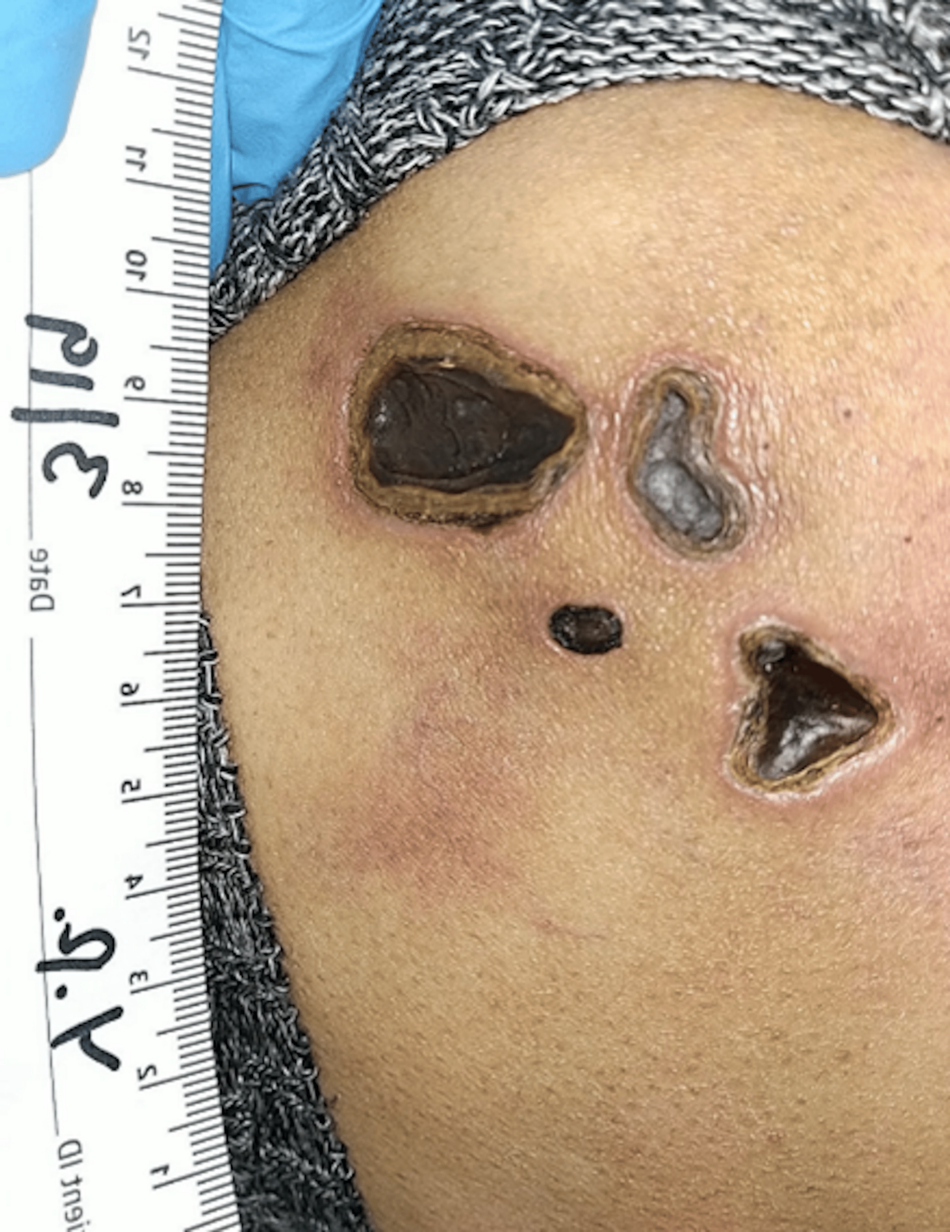
Initial wound presentation on March 19, 2024

**Figure 2:**
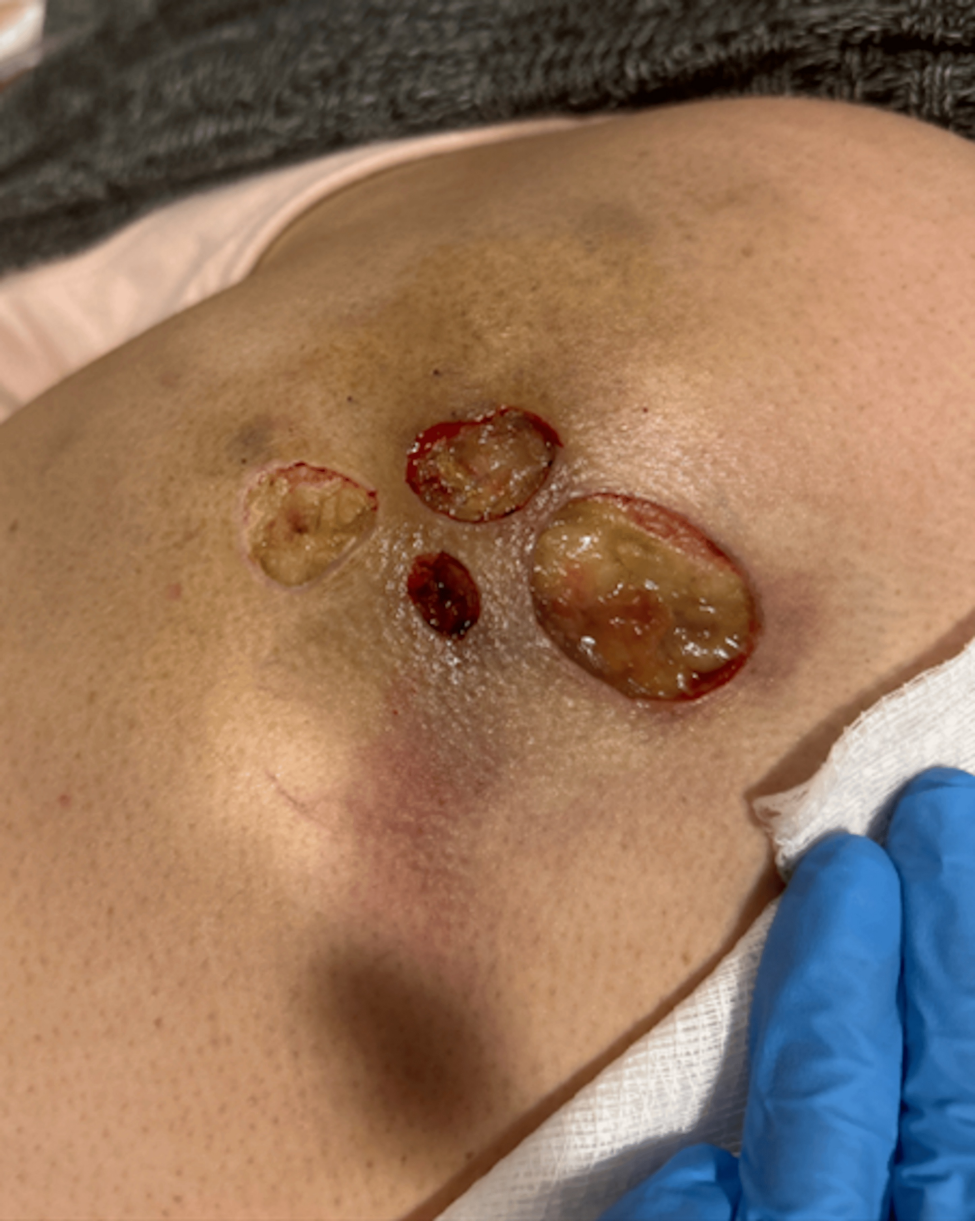
Wound presentation on March 19, 2024, s/p wound debridement

Upon admission, the wound had areas of necrosis, and the patient underwent wound debridement on March 19, 2024 (Figures 1, 2). The wound was then treated daily with Triad (Coloplast Corp., Minneapolis, MN, US) and active *Leptospermum *honey (MediHoney, Derma Sciences, Princeton, NJ, US) and dressed with wet-to-dry gauze soaked in hypochlorous acid (Vashe, Urgo Medical North America, Fort Worth, TX, US) solution, followed by the application of collagenase to enhance healing.

While hospitalized, the rheumatology team was consulted due to an arthritic flare-up. Upon review of the patient's home medications, sulfasalazine 2,000 mg was restarted and added to the continued prednisone regimen.

**Figure 3:**
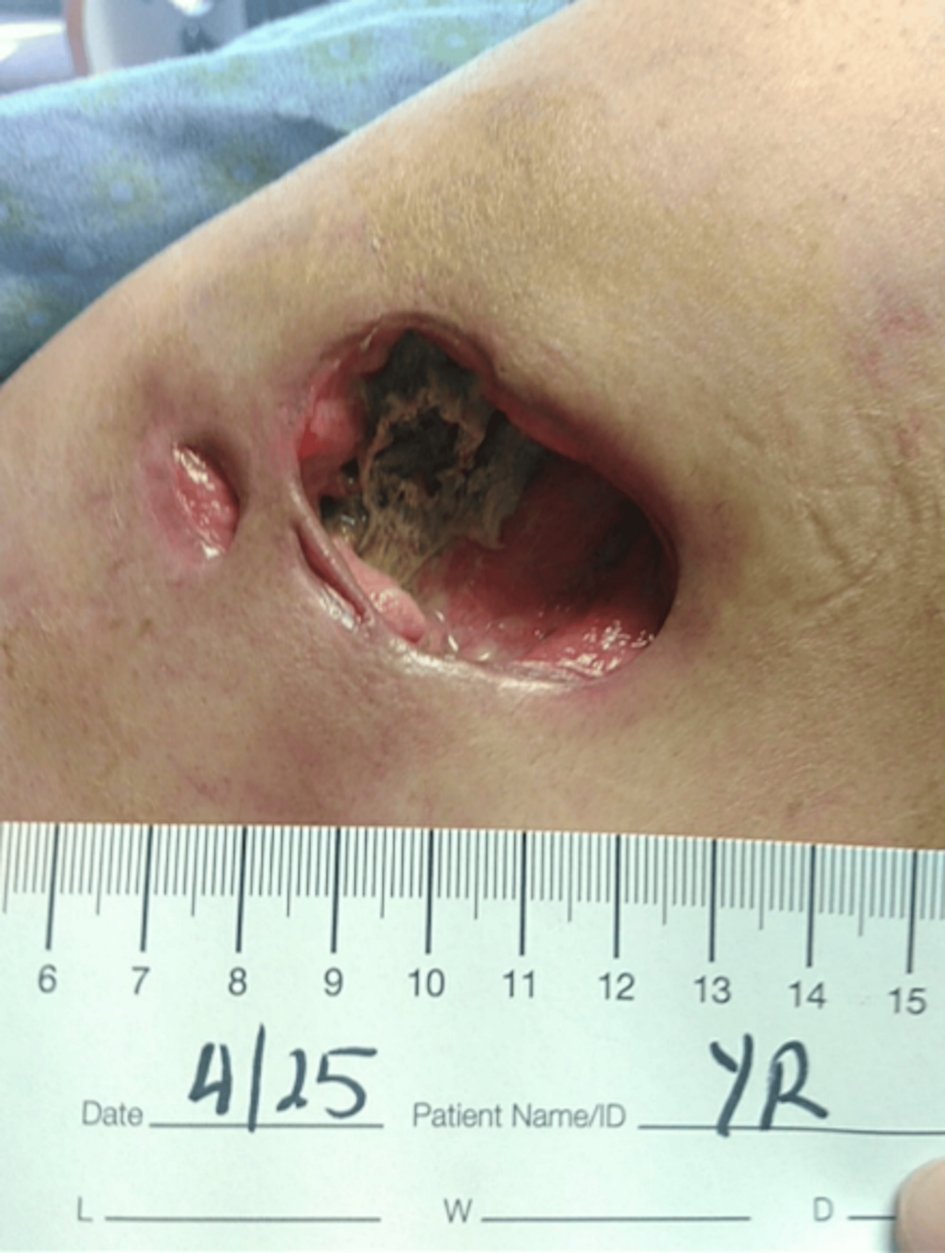
Wound presentation on April 26, 2024

During a follow-up on April 16, 2024, the wound appeared infected, and clindamycin was prescribed for 10 days. Nine days later (April 25, 2024), the patient returned to the hospital with an abscess within the wound, showing yellow-green discharge tunneling down to visible bone (Figure 3). The abscess was subsequently drained, revealing necrotic changes. The wound was managed initially with Vashe antimicrobial dressings every three days, which later progressed to daily application.

**Figure 4:**
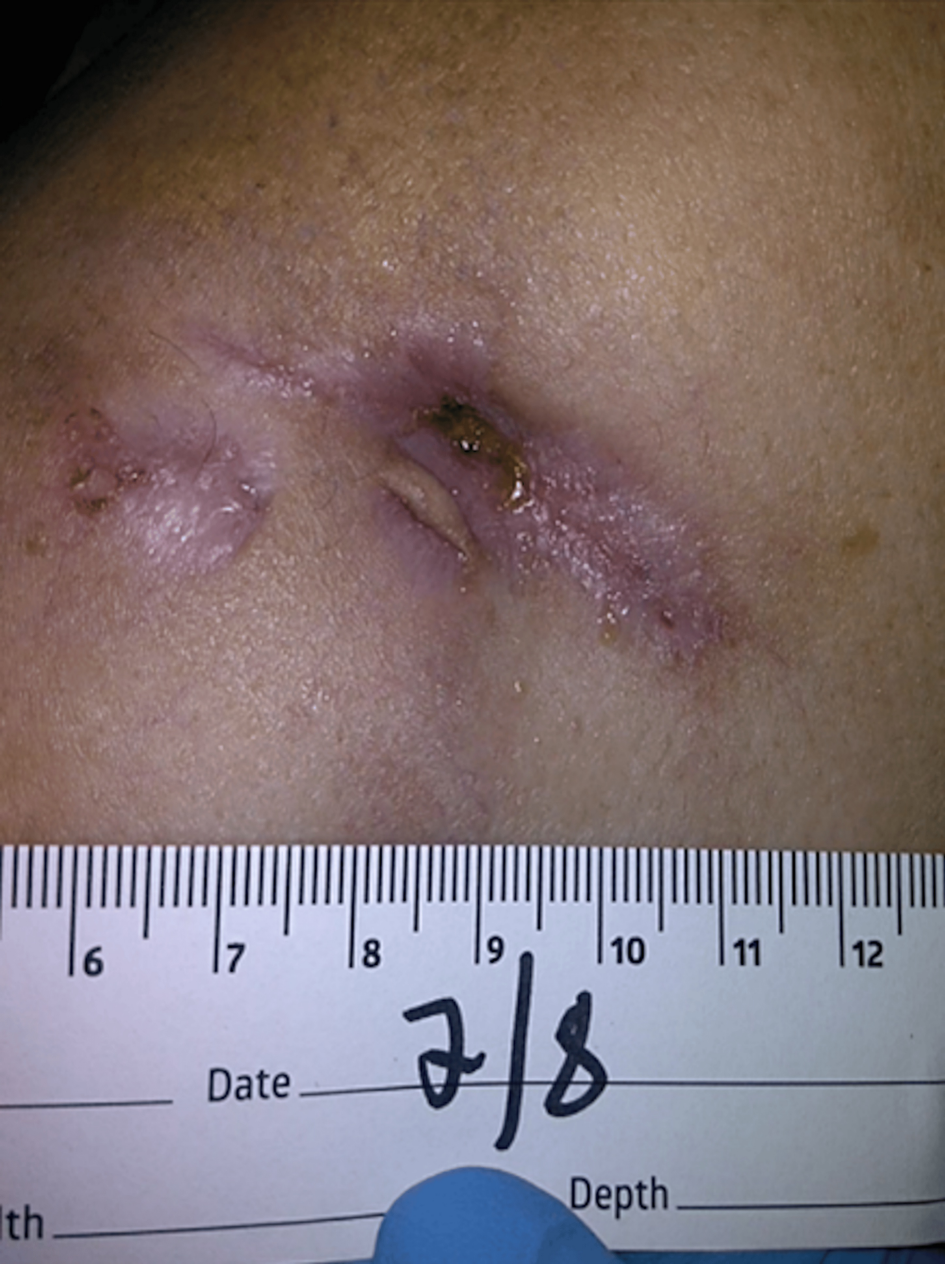
Wound presentation on July 8, 2024

At the final outpatient visit on July 8, 2024 (Figure 4), the wound demonstrated significant improvement and near resolution. It was cleansed with sterile normal saline, followed by the application of sodium carboxymethylcellulose (Aquacel, ConvaTec, Princeton, NJ, US) and foam dressings every other day.

## Discussion

In this patient, PG developed despite daily treatment with 5 mg of prednisone for RA. Although PG is classified as a sterile lesion, the patient’s chronic steroid use and T2DM likely increased her vulnerability to infection, culminating in the formation of an abscess and necrosis. Her multiple chronic conditions-particularly RA, a disease frequently associated with PG-coupled with limited financial resources further hindered her access to outpatient clinics and hospital care, complicating disease management.

This case is particularly notable due to the numerous barriers the patient faced in accessing recommended care. For instance, no dermatologist in the area accepted her insurance, and while infliximab was often suggested as an effective treatment [4], it was not covered by her plan. Additionally, the increased prednisone dose required more frequent clinic visits and adjustments to manage her T2DM. Fortunately, a low-income-friendly clinic played a pivotal role in her care, offering tailored medication adjustments, strict point-of-care glucose monitoring, and careful prednisone tapering during her recovery. Without access to this community clinic, the patient might have experienced preventable complications.

While the precise cause of PG in this patient is unclear, her history of RA and T2DM likely contributed to the rapid disease progression and heightened susceptibility to infection. These findings are consistent with the literature; a retrospective review of PG comorbidities reported that 19% of patients also had seronegative arthritis [5]. In our case, the patient’s treatment spanned approximately four months. Interestingly, studies have shown that patients with both PG and arthritis often require a longer treatment duration, averaging 14.8 months, compared to 8.7 months for PG patients without arthritis [6].

PG, in this case, was effectively managed using a regimen that included wound care, debridement, collagenase, prednisone, and sulfasalazine. Corticosteroids and sulfasalazine are widely recognized for their efficacy in treating PG. In a cohort of 34 patients, sulfasalazine achieved a complete response in 59% of cases, with 32% reporting no response [7]. Notably, 85% of patients in the study received sulfasalazine in combination with other therapies, most commonly glucocorticoids [7]. While corticosteroids remain the cornerstone of PG treatment, severe cases often necessitate the addition of biologics or immunosuppressants, which have yielded variable outcomes [8].

The role of surgical debridement in PG remains unclear. While some cases support its use, others report adverse outcomes, such as triggering new lesion formation [8,9]. However, evidence suggests that conservative wound debridement targeting necrotic tissue does not exacerbate existing PG wounds [9].

## Conclusions

This case underscores the importance of recognizing the presentation of PG and highlights an effective treatment strategy for a patient with multiple chronic comorbidities in a vulnerable, low-income community. The relatively simple and accessible treatment regimen used in this case aligns with established therapies for PG and emphasizes the critical need for regular follow-ups and stringent management of comorbid conditions to prevent complications and support recovery.
